# 4D Structural root architecture modeling from digital twins by X-Ray Computed Tomography

**DOI:** 10.1186/s13007-021-00819-1

**Published:** 2021-12-04

**Authors:** Monica Herrero-Huerta, Valerian Meline, Anjali S. Iyer-Pascuzzi, Augusto M. Souza, Mitchell R. Tuinstra, Yang Yang

**Affiliations:** 1 Institute for Plant Sciences, College of Agriculture, Purdue University, West Lafayette, IN USA; 2 Department of Botany and Plant Pathology, Purdue University, West Lafayette, IN USA

**Keywords:** Phenotyping, Imaging, Proximal sensing, 3D modeling, Skeleton, Root system architecture (RSA), X-ray CT (computed tomography), Digital twin

## Abstract

**Background:**

Breakthrough imaging technologies may challenge the plant phenotyping bottleneck regarding marker-assisted breeding and genetic mapping. In this context, X-Ray CT (computed tomography) technology can accurately obtain the digital twin of root system architecture (RSA) but computational methods to quantify RSA traits and analyze their changes over time are limited. RSA traits extremely affect agricultural productivity. We develop a spatial–temporal root architectural modeling method based on 4D data from X-ray CT. This novel approach is optimized for high-throughput phenotyping considering the cost-effective time to process the data and the accuracy and robustness of the results. Significant root architectural traits, including root elongation rate, number, length, growth angle, height, diameter, branching map, and volume of axial and lateral roots are extracted from the model based on the digital twin. Our pipeline is divided into two major steps: (i) first, we compute the curve-skeleton based on a constrained Laplacian smoothing algorithm. This skeletal structure determines the registration of the roots over time; (ii) subsequently, the RSA is robustly modeled by a cylindrical fitting to spatially quantify several traits. The experiment was carried out at the Ag Alumni Seed Phenotyping Facility (AAPF) from Purdue University in West Lafayette (IN, USA).

**Results:**

Roots from three samples of tomato plants at two different times and three samples of corn plants at three different times were scanned. Regarding the first step, the PCA analysis of the skeleton is able to accurately and robustly register temporal roots. From the second step, several traits were computed. Two of them were accurately validated using the root digital twin as a ground truth against the cylindrical model: number of branches (RRMSE better than 9%) and volume, reaching a coefficient of determination (R^2^) of 0.84 and a P < 0.001.

**Conclusions:**

The experimental results support the viability of the developed methodology, being able to provide scalability to a comprehensive analysis in order to perform high throughput root phenotyping.

**Supplementary Information:**

The online version contains supplementary material available at 10.1186/s13007-021-00819-1.

## Background

Plant roots are critical for water and nutrient uptake from soils [1, 2]. Roots can form complex networks composed by different type and age of roots [3]. The spatial arrangement of the root system is called Root System Architecture (RSA). Considering that RSA can affect crop performance, selecting crops based on specific RSA could lead to improve agricultural productivity [4]. However, our understanding of RSA development in soil is limited by the complexity of the root phenotyping in situ [5, 6]. Because of the opaque nature of soil, progress made in non-destructive root phenotyping has been limited to systems such as the rhizotron, which acquires two-dimensional images of root growing in transparent enclosures.

Plant science community urgently requires advance approaches in the characterization of RSA using novel image-based technologies [7], to quantify the 3D dynamics in RSA [8, 9]. Three tomographic techniques are currently available for non-destructive 3D phenotyping: X-ray Computed Tomography (CT), Magnetic Resonance Imaging (MRI) and Position Emission Tomography (PET). Recent technological innovations in scan resolution and the throughput in image processing made X-ray CT the current state of the art technology for non-destructive root phenotyping in soil [10]. Generally speaking, a regular X-ray CT has a source and a detector. The source is responsible for passing the X-ray beams through a sample, which absorbs a portion of these beams, while the detector will record this attenuated signal as two-dimensional projections. The attenuation is based on the material properties and electron-density; thus, the internal structure of the scanned sample becomes visible by contrasting the different elements inside depending on how much X-ray they absorb based on their chemical composition and characteristics [11]. Further, a 3D reconstruction of the sample material can be generated based on the 2D projections by scanning it at different positions [12].

A shape descriptor highly recommended in plant science is the curve-skeleton. It is able to describe the hierarchies and extent of branching plant networks [13]. Methods for skeleton extraction are primarily grouped in volumetric and geometrics, depending on the computed interior or only surface representation. As a common drawback, volumetric approaches potentially lose details and have numerical instability caused by inappropriate discretization resolution [14, 15]. In contrast, geometric methods approximate the medial surface by extracting the internal edges and faces. Medial axis skeleton and Reeb-graph-based methods are a couple of examples that are established using the geometric principles. In the 3D space, the medial axis usually fails when the planes occurrence.

For methods in 3D modeling, there are as well two categories. The first one includes voxel approaches, where volumetric models are constructed by partitioning the point cloud into voxels. The capability of these methods in model irregular surfaces is limited. The other category comprises parametric surface methods. The circular cylinder is the most dominant shape-fitting approach, because of its balancing between simplicity and realistic modeling [16].

Analysis of root models derived from X-ray CT images allows quantification of root growth over time and in response to external stresses, but there are several major challenges associated with this data. These include root segmentation and 3D modeling, which involve extracting the root digital twin from X-ray radiographs, and computing root architecture measurements from resulting models. The RootTine protocol was design to segment the root in a faster and automated way to be implemented in high-throughput (HTP) systems [17]. However, this method only computes the root length as a phenotyping trait by medial axis-based skeletonization processes. RootForce [18] is one of the latest developments in semi-automatic segmentation based as well in RootTine. Therefore, an initial phase is required to tuned these parameters on few samples. Once these parameters are adapted to the pot, soil and root system, the same set of parameters can be used for a complete time series experiment. Based on these arguments, RootFroce is described as especially designed for highthrouput time series of CTX data. It is able to extract more traits, for instance root volume and root growth angles by Reeb Graph-based skeletonization. RooTrack is another tool for not only root segmentation but also for visually object tracking by identifying boundaries in image cross-sections. The main advantage is detecting and differentiating multiple roots from different plants in the same image. Still, this methodology is not yet applicable to HTP or automated procedures [19]. These tools mainly tackle the root segmentation issue from X-ray data as a primary challenge. The focus of this paper is to model temporal digital twins of roots to quantify traits as well as to record the topological and hierarchical branching structure, after the segmentation from the soil is already done. To the best of our knowledge, no research has been done to parametrize by geometric primitives the root surfaces or even label their different branches using a volumetric model. In our methodology, the temporal analysis of roots is solved throw skeleton extraction, while the spatial quantification is performed by a shape-fitting approach.

In this paper, we propose a spatial–temporal root architectural model from digital twins obtained by X-ray CT (computed tomography). Values of essential root traits were extracted as phenotypic data to quantitatively assist growth analyses and RSA description. The proposed methodology consists of two phases. In the first, we compute a curve-skeleton as a powerful descriptor for analyzing root system networks. We use a constrained Laplacian smoothing algorithm which directly performs on the mesh domain, followed by a connectivity surgery and embedding refinement process. As a result, this skeletal structure controls the registration process in temporal series. Secondly, the root system is robustly reconstructed by generating a flexible cylinder model. This non-linear optimization problem is solved by nonlinear squares iterative solution. The full pipeline is optimized for quantifying accurate and robust results, allowing high-throughput root phenotyping using X-ray CT systems.

## Materials and methods

### Materials

The 3D digital twin of the root system is obtained by X-ray CT. This technology allows us to non-destructively, comprehensively and accurately monitor the exact same plant root even at different points in time under controlled conditions. Our system scans pots with photon energies in the 225 keV range, and is able to scan pots 20 cm in height in less than 7 min. The resulting voxel size is set at 200 μm. The Focus-Detector distance is 800 mm. Both X-ray detector and X-ray tube are fixed within the system. A pot rotation stage allows 360° for the measurement. A vertical translation axis optionally extends the vertical field of view. Table 1 summarizes the rest of the technical specifications of the system. The system manufacturer is Fraunhofer IIS (Fraunhofer Development Center X-ray Technology, Germany).

**Table 1:** Technical specifications of the X-ray CT system

	Parameter		Value
X-ray cabin		Pot diameter	100–200 mm
		Pot height	≤ 400 mm
		Footprint dimension	2500 * 1500 * 3500 mm^3^
		Sample height	≤ 2500 mm
		Weight Plant weight	≤ 6 ton ≤ 7500 g
X-ray source		Max tube voltage Max tube power at small focal spot	225 kV 800 W
		Used voltage Used current	200 kV 3.5 mA
		Used power Cooper filter	700 W 1 mm
		Cooling device	Included
Detector system		Type	Xeye 2530 flat panel detector Radiation hard detector
		Size	300 * 250 mm^2^
		Pixel matrix	3333 * 2777
		Pixel pitch	90 µm

**Fig. 1:**
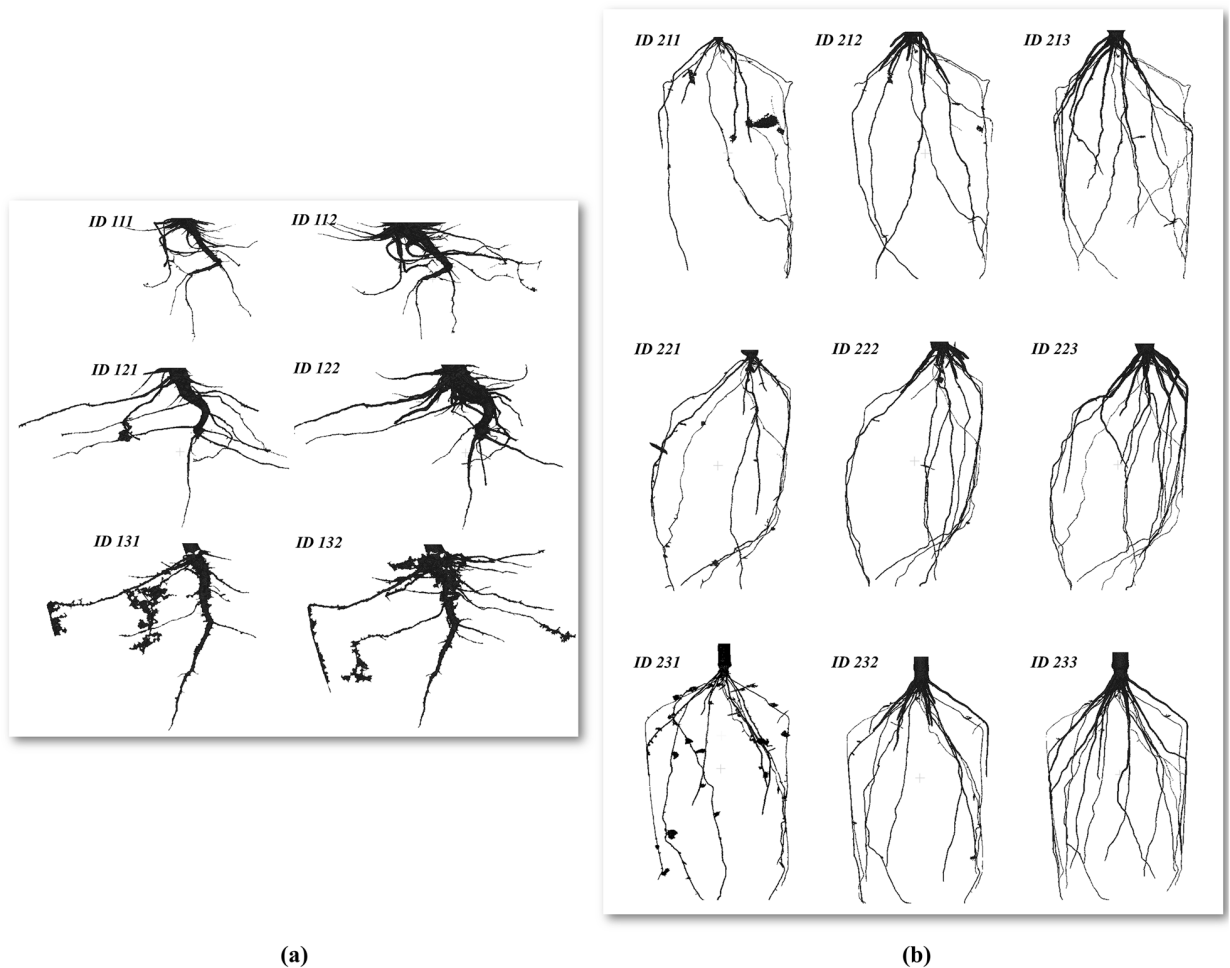
Visualization of the root samples from tomato (**a**) and corn (**b**) used in this study

The experiment was performed at the Ag Alumni Seeds Phenotyping Facility (AAPF) at Purdue University in West Lafayette (IN, USA). In this facility, plants are transported in standard carriers to the X-ray CT system from the loading position by a mechanical conveyor belt. During the summer of 2019, root systems from three tomato plants at two different times and three corn plants at three different times were scanned. The pots were circular with 180 mm-diameter and 200 mm-height for tomato and 400 mm-height for corn. The type of pot media in the pots is sifted sphagnum peat moss with a moisture inferior at 20% of relative humidity. Table 2 summarize the main characteristics of the digital twins of the roots used in this study (additional file as Data S1: dataset), whereas Fig. 1 shows their visualizations.

**Table 2:** Root digital twin dataset

	Sample	Scanned date	Scan ID	Num. vertices	Num. faces
Tomato	11	July 2nd, 2019	111	120468	240928
		July 18th, 2019	112	252550	505148
	12	July 3rd, 2019	121	183968	367920
		July 18th, 2019	122	361248	722628
	13	July 9th, 2019	131	110899	224002
		July 23rd, 2019	132	427124	854604
Corn	21	July 9th, 2019	211	688928	1378152
		July 15th, 2019	212	834648	1669464
		July 23rd, 2019	213	1106486	2213120
	22	July 9th, 2019	221	729842	1459864
		July 15th, 2019	222	935870	1871920
		July 23rd, 2019	223	1211190	2422576
	23	July 9th, 2019	231	765291	149761
		July 15th, 2019	232	938634	1877412
		July 23rd, 2019	233	1207404	2414924

### Methodology

**Fig. 2:**
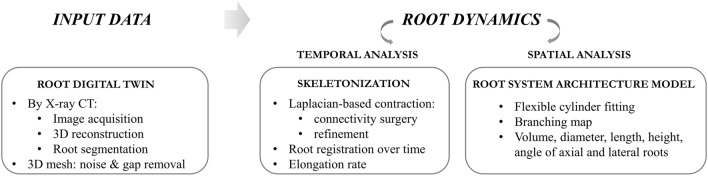
Workflow of the methodology proposed: from the digital twin of the root, first we extract the curve-skeleton to register temporal series of the same root and secondly, we spatially model the RSA by a flexible cylinder fitting

In this study, we developed an approach that can be used to enable high throughput root phenotyping tasks. It includes a 4D structural root architectural modeling from digital twins. These digital twins were acquired X-ray CT using RootForce tool [18]. RootForce approach is based on Frangi’s vesselness method [20], extended for the semi-automatic segmentation of roots. Beforehand, a thresholding is applied to select a range of attenuation coefficient according to the type of soil and plants used in the experiment. Then, the Hessian-based Frangi vesselness filter is used for small roots detection while larger roots are detected based on their 3D homogeneity using a 3D-Gaussian filter. The small and large vessel structures are then merged using upper and lower merging thresholds. Here, the value range of the attenuation coefficient was 0.07 to 0.19 with root diameters of 0.4, 0.5, 0.6, 1.0 and 1.2 mm. The upper and lower threshold of the merging parameters were respectively 25 and 1000 for the corn roots and respectively 100 and 1000 for the tomato roots. A size filter was used to eliminate unconnected fragment with a minimum volume of 25 mm^3^ for the corn roots and 50 mm^3^ for the tomato roots. The minimum root diameter that can be segmented with RootForce is about 2.5 voxels in diameter. Here, using a resolution of 200 μm cubic voxel size for the reconstruction, the minimum detectable root diameter is approximatively 0.5 mm. Once the segmentation process is done, we apply our model approach. It consists of two clearly differentiated phases: the computation of the curve-skeleton which serves for the registration of temporal series, and the RSA cylindrical model of the digital twin for spatial analysis. Figure 2 summarizes the workflow to follow.

### Skeletonization

Basically, the curve-skeleton is a structure that extracts the volume and topological characteristics of the model. We select a robust skeleton extraction method via Laplacian-based contraction [14, 15] based on the characteristics of the model: the algorithm works directly on the mesh, without a resampled volumetric representation. By this means, it is pose-insensitive and invariant to global rotation. As a potential limitation of this skeleton algorithm, it only works for closed mesh models with manifold connectivity since the Laplacian contraction algorithm operates for every individual vertex. In order to close the mesh, we follow the procedure already explained by [21], which incorporates several automatic and sequential tasks: (i) filling of holes through algorithms based on interpolators of radial basis function [22]; (ii) repairing of meshing gaps by threshold distance algorithms [23]; (iii) removing of topological noise, allowing the mesh to be re-triangulated locally [24]; (iv) removing of topological and geometric noise by anti-aliased Laplacians filters [25]. Once the mesh is closed, the skeleton extraction is applied. Firstly, the method contracts the mesh geometry into a zero-volume skeletal shape. Details and noise are removed by applying an iterative Laplacian smoothing that tightly moves all the vertices along their curvature normal directions. After each iteration, a connectivity process is carried out, removing all the collapsed faces from the degenerated mesh until no triangles exist. The key of this step is to sensibly control the contraction procedure so that it leads to a collapsed mesh with sufficient skeletal nodes to maintain an acceptable correspondence between the skeleton and the original geometry. As a consequence, the contraction does not alter the mesh connectivity and retains the key features, guarantying to be homotopic to the original mesh. Next, we describe a process that moves each skeletal node to the center of mass of its local mesh region in order to refine the skeleton’s geometric embedding.

This skeletal structure drives the registration process in temporal series. Thus, we can automatically perform a growth analysis of the RSA, quantified by the elongation rate as a trait. To register temporal series, Principal Component Analysis (PCA) is performed [26]. In general, the principal components are eigenvectors of the data's covariance matrix. More specifically, this statistical analysis uses the first and second moments of the curve-skeleton, resulting in three orthogonal vectors centered on its center of gravity. The PCA summarizes the distribution of the lines along the three dimensions and models the principal directions and magnitudes of the curve-skeleton distribution around the center of gravity. Thereby, the registration of temporal series is carried out by overlapping the principal component axes. The elongation rate is measured in the first principal direction.

### RSA model

We use a group of geometric primitives to model the surface and topology of the root. The circular cylinder is the simpler primitive. For natural entities such as trees, the circular fitting is the most robust primitive in the sense of a well-bounded volumetric modelling error, even with noise and gaps in the data, compared with more complex primitives which are more sensitive to data quality [16]. Thereby, our modeling is based on circular cylinder fitting as an optimal parametrization to provide significant traits of the RSA such as diameters, specific surfaces and volumes from the main root and ramifications. We use the approach of [27], where they model point clouds of individual trees acquired from TLS (Terrestrial LiDAR Scanner) by a cylindrical parametrization. This process is scale independent because only neighbor-relations and relative sizes are needed. To apply this approach, the 3D mesh of the root digital twin is transformed into a regularized point cloud [28]. For that, randomly sampled points over the mesh are extracted by fixing a desired density, (5 points/mm^2^) and a restored point cloud is obtained. Subsequently, we apply Dart Throwing Poisson Disk sampling to the point cloud to make the points appear more uniform by culling those points that are close to a randomly selected point [29]. In this step, a threshold based on Euclidean distance between points of 1 mm is set. These values are set regarding the details in the final cylindrical model and due to the scanner’s accuracy for these specific samples. After this process, a significant reduction of points is achieved because the Poisson subsampling approach considers the local point distribution, retaining key elements of the structure.

Once the regularized point cloud is achieved, the cylinder fitting is applied. The process has 2 consecutive phases: first, the point cloud is segmented into the main root and its ramifications, and secondly, the surface and volume of the segments are robustly fitted with geometric primitives, specifically cylinders. This non-linear optimization problem is solved by nonlinear squares iterative solution. The topological distribution of the RSA is also recorded. Mathematically, the model is raised by a local approach in which the point cloud is covered with small sets corresponding to connected surface patches in the root surface. In that way, the RSA and size properties, such as volume and branch size distributions, can be approximated. The method uses a cover set approach [27], where the point cloud is partitioned into small sets that correspond to small patches in the surface of the model. These sets form the smallest unit we use to segment the point cloud into main root and individual branches. The generation process produces a Voronoi partition of the point cloud so that the cell size is controlled. The cover set value is calculated by an iterative approach where the final value varies from 0,75 to 3 cm.

## Experimental results

**Fig. 3:**
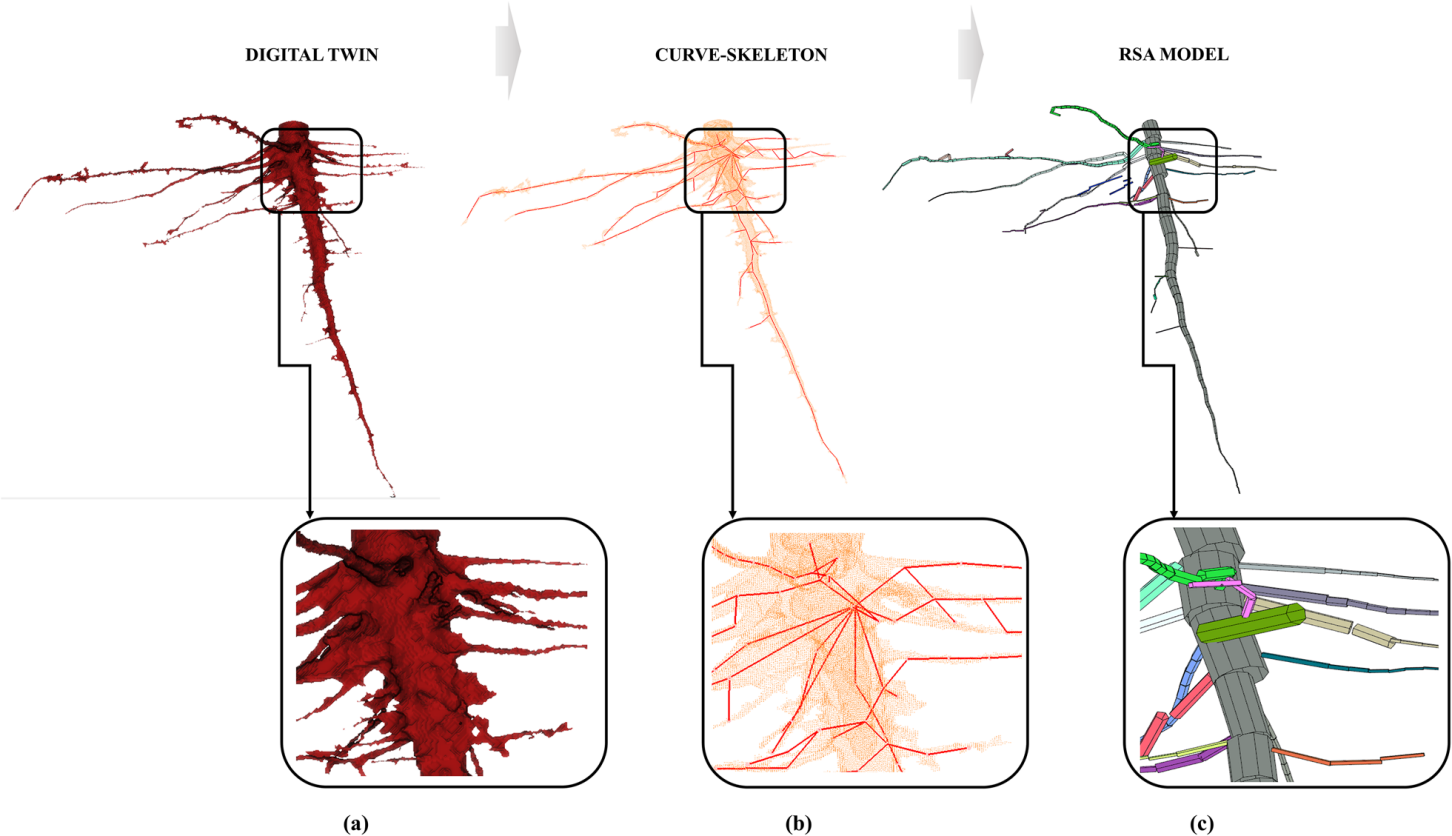
Tomato root sample with a zoom window: digital twin by X-ray CT system (**a**), curve-skeleton extraction based on a constrained Laplacian smoothing algorithm, where the mesh is in orange and the skeleton is in red (**b**), and the RSA model based on a flexible cylinder fitting, where each ramification is in different color (**c**)

**Fig. 4:**
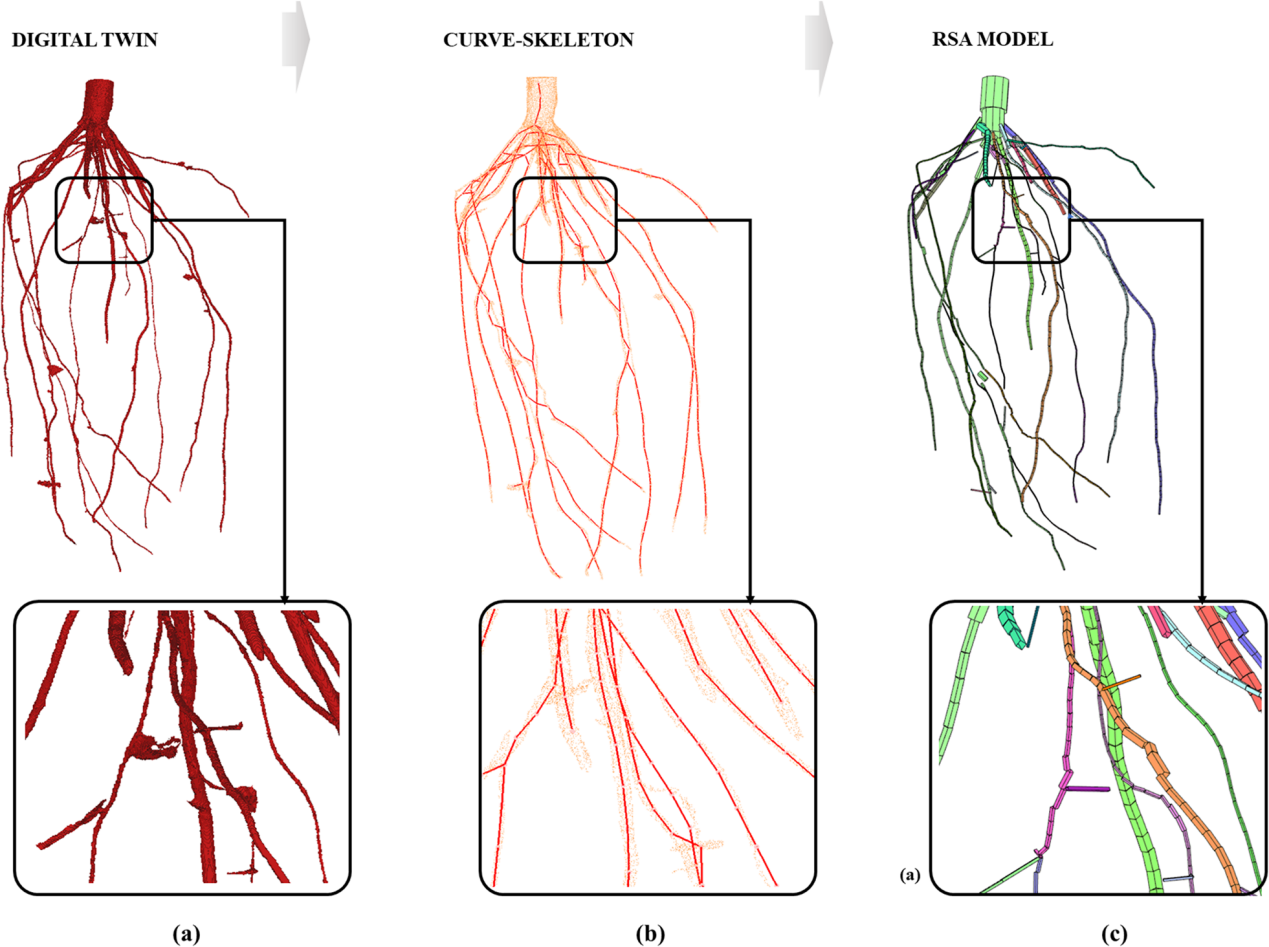
Corn root sample with a zoom window: digital twin by X-ray CT system (**a**), curve-skeleton extraction based on a constrained Laplacian smoothing algorithm, where the mesh is in orange and the skeleton is in red (**b**), and the RSA model based on a flexible cylinder fitting, where each branch is in different color (**c**)

All the experimental results obtained below were run on a 3.6-GHz desktop computer with an Intel CORE I7 CPU and 32-GB RAM. First, the digital twin of the root obtained by X-ray must be previously closed and repaired to be able to apply our approach as Sect. 2.2.1. explains. Once the mesh is closed, the skeleton extraction and the RSA model pipelines are run. The code from the RSA model saves (i) general values of the entire root as total volume, height, length, number and order of branches, and the mean and maximum diameter of the crown, (ii) branching map of the root that includes the topological relation of each ramification, (iii) volume, length, angle, height, azimuth and zenith of each branch, and (iv) length, diameter, angle and coordinates of all the cylinders that belong to each branch. Figures 3 and 4 show both results for a tomato and a corn root sample. In the zoom window, we can appreciate the complexity and accuracy of the model. In our RSA model, each branch is labeled in a unique color and quantified. This is a brand-new solution that is able to quantify branching patterns, which are critical for biologists to understand water and nutrient uptake. In the additional file 1, we made a video that shows the segmented root, the skeleton and the RSA model, for tomato and for corn (Additional file 2: Video S2: 4D Structural Root Architecture Modeling).

From the RSA model, different traits are extracted. Table 3 summarize the general values of the entire roots.

**Table 3:** General values of the RSA model for each sample (volume, volume of the main root, total length, length of the main root, number of branches, maximum order of ramifications and maximum and mean crown diameter)

Scan ID	Vol. (mm^3^)	Vol. Main Root (mm^3^)	Total Length (mm)	Main Root Height (mm)	Num. Branches	Max. order Branches	Crown Diam. Max (mm)	Crown Diam. Mean (mm)
111	1072.26	734.52	79.07	3.74	22	4	5.24	3.17
112	2107.96	948.02	138.87	3.82	31	4	6.66	4.71
121	735.04	767.75	120.18	4.43	22	4	7.13	4.84
122	3543.38	1847.13	172.55	4.73	35	4	7.42	5.19
131	1406.26	987.31	78.58	5.59	28	3	6.14	4.12
132	3759.36	2292.33	158.38	5.60	33	4	9.28	5.77
211	5505.33	1430.63	250.84	6.30	21	3	3.63	2.41
222	12,130.01	3772.18	313.17	6.47	22	3	3.65	2.88
223	15,935.05	5588.36	461.88	6.56	23	3	3.64	3.17
221	8119.29	3753.43	288.57	6.16	17	4	3.45	3.10
222	13,821.25	4615.14	346.23	6.43	22	3	3.55	3.19
223	17,448.18	4382.53	464.75	6.42	26	2	3.58	3.39
231	10,869.41	6765.38	308.37	6.27	20	3	3.57	2.97
232	17,793.40	9786.01	385.29	6.38	24	4	3.67	2.96
233	21,273.69	11,858.14	527.83	6.41	26	5	3.66	3.04

## Validation results and discussion

The volume of each digital twin of the root is measured by Cloud Compare software [30], that computes the volume within the solid mesh. Moreover, number of branches from digital twins are estimated by a visual analysis. Table 4 shows several metrics between the digital twin and the cylindrical model of each root of these two parameters. In particular, the root mean square error (RMSE), the relative RMSE (RRMSE), the average systematic error (ASE), and mean percent standard error (MPSE) were calculated as follow:

1 $${\text{RMSE}} = \sqrt {\frac{{\mathop \sum \nolimits_{{{\text{i}} = 1}}^{{\text{n}}} \left( {{\text{y}}_{{{\text{model}}}}^{{\text{i}}} - {\text{y}}_{{\text{dig twin}}}^{{\text{i}}} } \right)^{2} }}{{\text{n}}}}$$

2 $${\text{RRMSE}} = 100{*}\frac{{{\text{RMSE}}}}{{{\overline{\text{y}}}_{{\text{dig twin}}}^{{}} }}$$

3 $${\text{ASE}} = \frac{100}{{\text{n}}}{*}\mathop \sum \limits_{{{\text{i}} = 1}}^{{\text{n}}} \left( {{\text{y}}_{{{\text{model}}}}^{{\text{i}}} - {\text{y}}_{{\text{dig twin}}}^{{\text{i}}} } \right)/{\text{y}}_{{\text{dig twin}}}^{{\text{i}}} { }$$

4 $$MPSE = \frac{100}{n}*\mathop \sum \limits_{i = 1}^{n} |\left( {y_{model}^{i} - y_{dig twin}^{i} } \right)/y_{dig twin}^{i} |$$

**Table 4:** Statistic metrics of number of branches and volume where RMSE is the root mean square error, RRMSE is the relative RMSE, ASE is the average systematic error, and MPSE is the mean percent standard error

Parameter	Root	RMSE	RRMSE (%)	ASE (%)	MPSE (%)
# branches [number]	Tomato	2.48	8.76	1.37	1.67
	Corn	1.76	8.31	5.94	0.44
Total volume [mm^3^]	Tomato	1087.66	36.92	-29.01	0.48
	Corn	4470.03	25.11	-23.63	2.86

where *y*^*i*^_*model*_ is the parameter estimated from the model of the i^th^ scan, *y*^*i*^_*dig twin*_ is the measured parameter from the digital twin of the i^th^ scan, $\overline{y}_{{dig{ }twin}}^{{}} { }$ is the mean of the measured parameter from the digital twin per scan, and *n* is the number of scans.

**Fig. 5:**
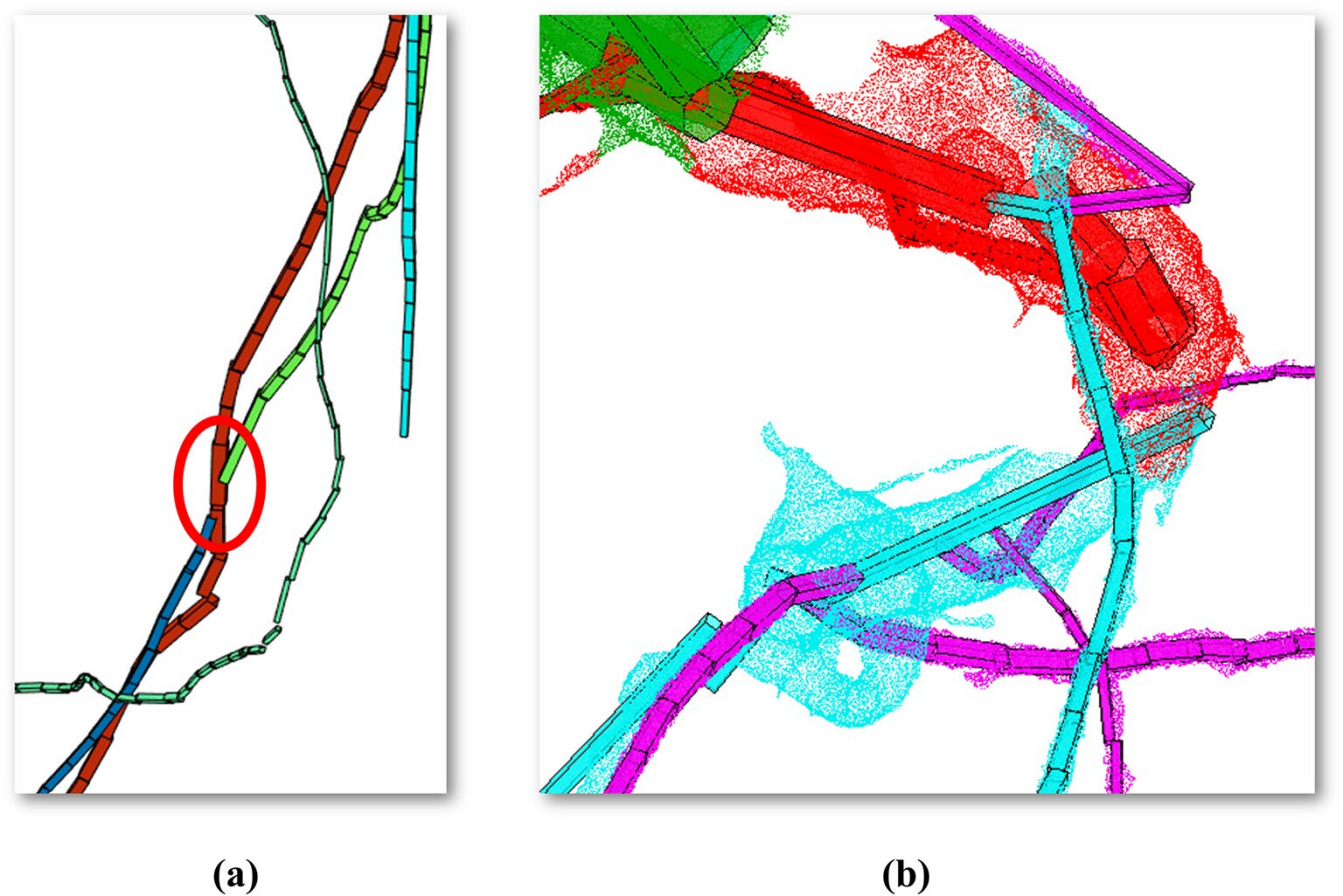
Errors in the number of detected branches due to loss of tracking in the segmentation process. Branches are on different colors with a red circle remarking this issue (**a**); and volume discrepancies in between the digital twin, represented with dense points, and the RSA model represented with polyhedrons with similar colors (**b**)

From the results of this table, we can affirm that our model detects branches mainly by excess in tomato and corn. In addition, for tomato branches were estimated by deficit more than for corn. Regarding the absolute volume discrepancies, they are larger for corn. In relative volume quantity, the error in tomato is larger. For both, always the volume is estimated by deficit. The errors in the number of branches detected could have been caused by segmentation problems. Figure 5a represents a part of the RSA model from the ID 121 tomato scan. Each detected branch is in a distinct color. We can see that the loss of the tracking of the branches could have generated new false branches. This issue is high-lighted with a red circle in the figure. Another type of common errors is the volume discrepancies between the digital twin and the RSA model, mainly generated when the shape of the branch is not cylindrical and when the diameter of each segmented branch does not decrease along the length. This topological property is used in the branch segmentation of the model. Figure 5b represents a part of the ID 223 corn scan, where the digital twin is represented by points and the model by polyhedrons in the same color. The shape of the branches could generate errors to fit cylindrical solids.

**Fig. 6:**
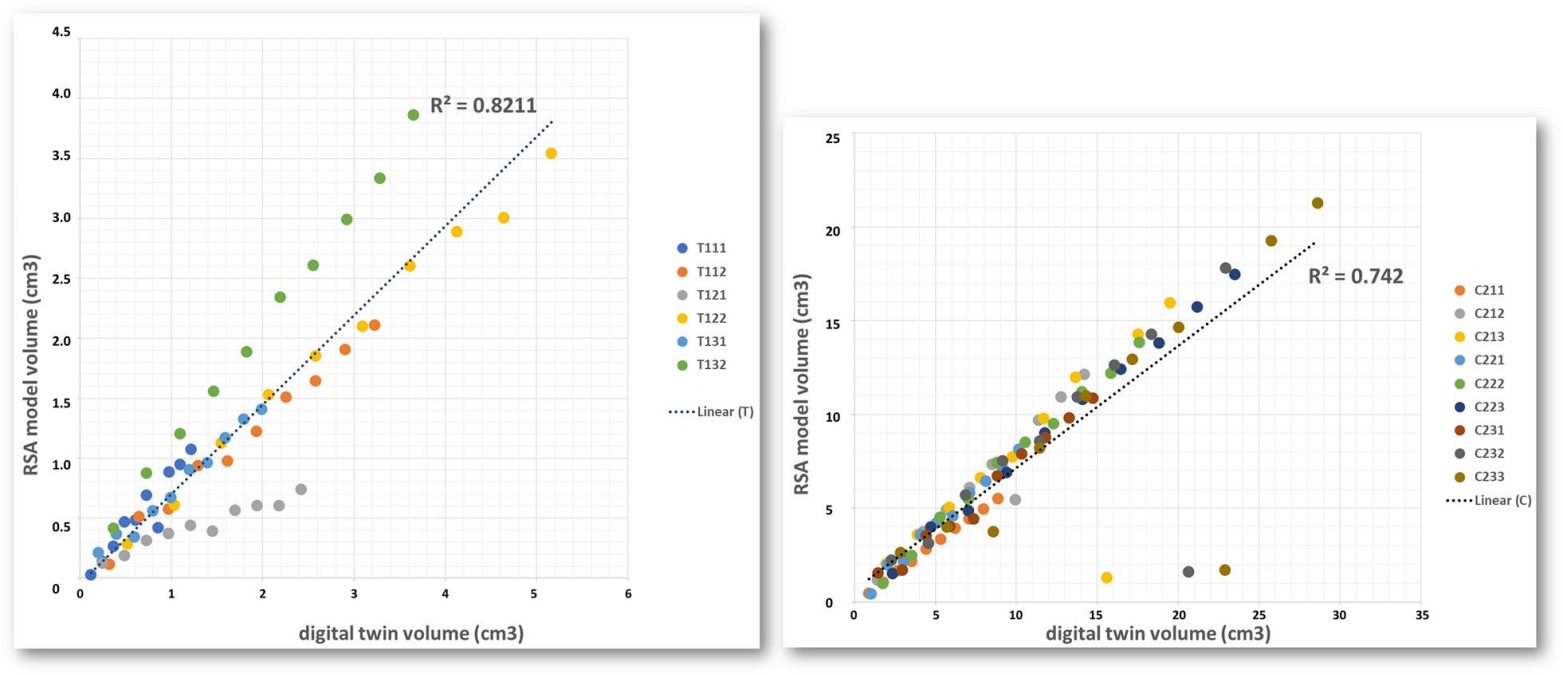
Volume correlation between the RSA model and the digital twin for each scan sample: tomato (**a**) and corn (**b**)

The relative volume of the RSA model is compared against the relative volume from the digital twin measured by Cloud Compare software [30]. We split the digital twins in 10, 20, 30, 40, 50, 60, 70, 80 and 90% of the total volume (starting from the top) and we run the model with these parts to evaluate its performance, which reached an R^2^ of 0.82 for tomato and 0.74 for corn with a P < 0.001, as Fig. 6 displays. When tomato and corn measurements are together, the R^2^ improves to 0.83.

**Fig. 7:**
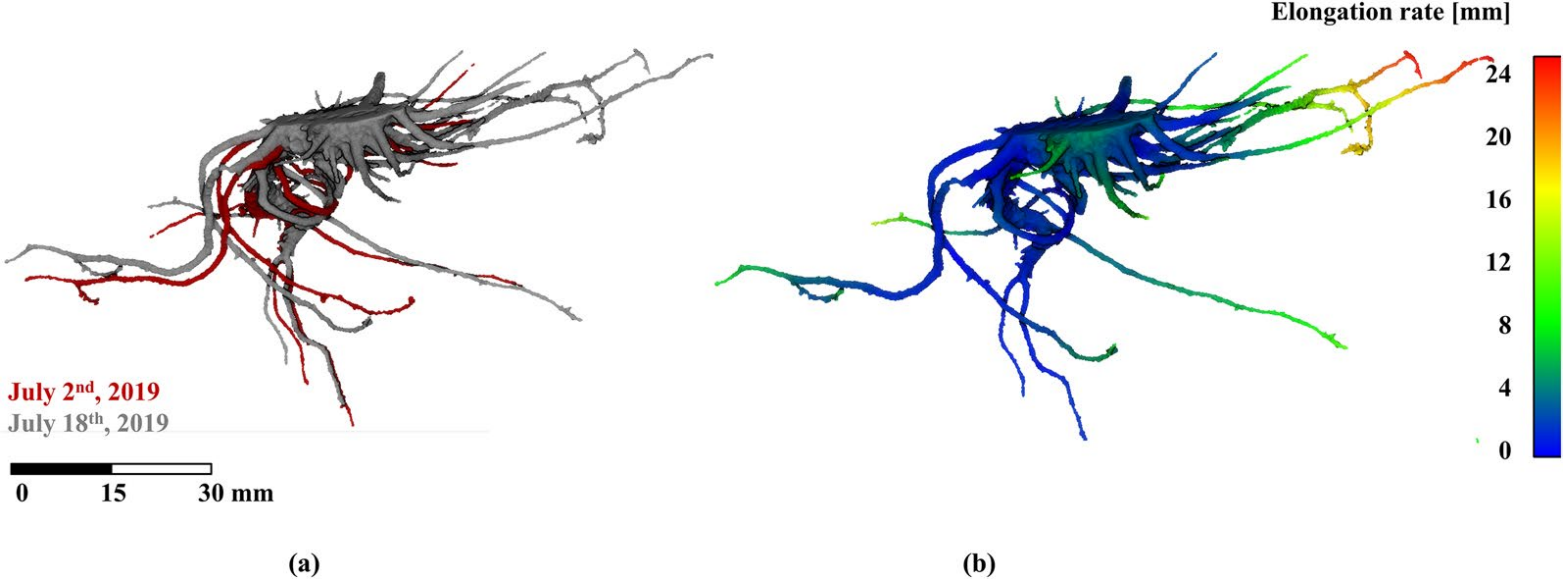
Tomato root sample in July 2nd and 18th, 2019, registered by a PCA of the skeleton (**a**) and the elongation rate mapped in 3D (**b**)

**Fig. 8:**
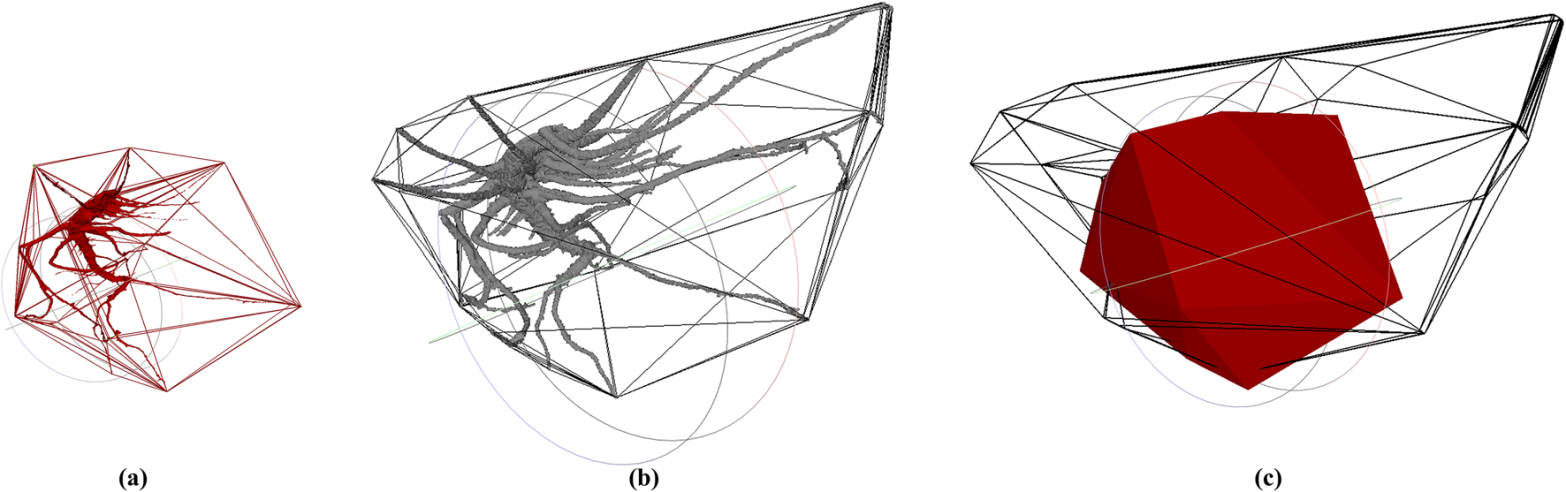
Mesh of the tomato root sample from July 2nd (**a**) and 18th (**b**), together with its convex hull. Both convex hulls registered and superimposed in solid and transparent faces (**c**)

Furthermore, this methodology is able to temporally analyse the root dynamics through a registration process based on a PCA of the skeleton from the root mesh. Figure 7a shows the same tomato root sample registered at two different times (July 2nd and 18th, 2019), with a slot of 16 days. The elongation rate is mapped in Fig. 7b, where the maximum value is 2.58 cm on the upper-right ramification. Figure 8 illustrates the same temporal sample where the convex hull is individually computed (Fig. 8a, b) and as well the variation in time (Fig. 8c). The convex hull value for Fig. 8a is 229.87 cm^3^ and for Fig. 8b is 519.76 cm^3^. At this point, it is worth to notice that PCA results are affected by the segmentation process: the better segmentation is done, the more accurate PCA results are obtained.

Table 5 recaps the maximum and mean value of the elongation rate for the temporal series of each sample and the convex hull volume reached by each root.

**Table 5:** Values of the volume of the convex hull and maximum and mean elongation

Scan ID	CH Volume (cm^3^)	Max. elongation (cm)	Mean elongation (cm)
111	2298.71		
112	5197.62	2.58	0.63
121	6183.22		
122	15,525.64	3.02	0.97
131	5762.72		
132	9502.78	1.98	0.62
211	50,631.14		
212	64,718.84	5.23	1.07
213	64,131.16	4.02	0.76
221	58,427.65		
222	58,146.56	3.75	0.98
223	63,539.13	4.14	0.77
231	61,435.67		
232	62,898.37	4.01	0.87
233	63,957.62	3.89	0.79

## Conclusions

To sum up, the developed pipeline aims to automatically extract phenotypic data of RSA from digital twins obtained by non-invasive X-ray CT. This pipeline is able to analyze both spatial and temporal root dynamics. As potential advantages, we find this methodology fully automatic, fast, precise and sufficiently robust to provide scalability for high throughput root phenotyping.

Determining the contribution of structural root traits to crop performance is vital to overcome climate change, environmental degradation and food insecurity. In addition, structural root traits that are accurately extracted from X-ray data will enhance our understanding of the relationship between the plant phenome and plant function in ecosystems, which is the end goal of functional phenomics [31]. Moreover, this computationally low-cost workflow will potentially increase the usability of imaging technologies for high-throughput phenotyping regarding genetic mapping and phenotypic selection in breeding programs.

## Supplementary Information

**Additional file 1.** Dataset.

**Additional file 2.** 4D Structural Root Architecture Modeling.

## Data Availability

The dataset supporting the conclusions of this article is included within the article (Additional file 1: Data S1: dataset and Additional file 2: Video S2: 4D Structural Root Architecture Modeling).
